# Heat-Modified Citrus Pectin Induces Apoptosis-Like Cell Death and Autophagy in HepG2 and A549 Cancer Cells

**DOI:** 10.1371/journal.pone.0115831

**Published:** 2015-03-20

**Authors:** Lionel Leclere, Maude Fransolet, Francois Cote, Pierre Cambier, Thierry Arnould, Pierre Van Cutsem, Carine Michiels

**Affiliations:** 1 Laboratory of Biochemistry and Cellular Biology-URBC, NARILIS, University of Namur, 61 rue de Bruxelles, 5000, Namur, Belgium; 2 Laboratory of Plant Cellular Biology-URBV, University of Namur, 61 rue de Bruxelles, 5000, Namur, Belgium; University of Pittsburgh, UNITED STATES

## Abstract

Cancer is still one of the leading causes of death worldwide, and finding new treatments remains a major challenge. Previous studies showed that modified forms of pectin, a complex polysaccharide present in the primary plant cell wall, possess anticancer properties. Nevertheless, the mechanism of action of modified pectin and the pathways involved are unclear. Here, we show that citrus pectin modified by heat treatment induced cell death in HepG2 and A549 cells. The induced cell death differs from classical apoptosis because no DNA cleavage was observed. In addition, Z-VAD-fmk, a pan-caspase inhibitor, did not influence the observed cell death in HepG2 cells but appeared to be partly protective in A549 cells, indicating that heat-modified citrus pectin might induce caspase-independent cell death. An increase in the abundance of the phosphatidylethanolamine-conjugated Light Chain 3 (LC3) protein and a decrease in p62 protein abundance were observed in both cell types when incubated in the presence of heat-modified citrus pectin. These results indicate the activation of autophagy. To our knowledge, this is the first time that autophagy has been revealed in cells incubated in the presence of a modified form of pectin. This autophagy activation appears to be protective, at least for A549 cells, because its inhibition with 3-methyladenine increased the observed modified pectin-induced cytotoxicity. This study confirms the potential of modified pectin to improve chemotherapeutic cancer treatments.

## Introduction

Cancer remains one of the leading causes of death worldwide. Despite a wide range of therapeutic approaches, cancer cannot be easily cured, and many cancer types still have a low cure rate. Although essential for treatment, chemotherapy and radiotherapy are also sources of many side effects, and surgery can sometimes miss metastases. These are reasons why the development of new therapies to improve existing treatments is a major challenge. Natural compounds and phytochemicals have recently attained much interest for their ability to modulate the signaling pathways involved in cancer proliferation and metastasis or for their protective potential in radiotherapy, as reviewed by Hazra [1].

Pectins are abundant and complex components of the primary plant cell wall and are well known as dietary fiber. Pectin polysaccharides include homogalacturonan (HG), substituted galacturonans, rhamnogalacturonan-I (RG-I) and rhamnogalacturonan-II (RG-II). HG is a polymer of α-1,4-linked-D-galacturonic acid, and HG residues can be methyl-esterified at the C-6 carboxyl or acetylated at the O-2 or O-3, depending on the pectin source. The backbone of HG is covalently cross-linked to RG-I and RG-II. RG-I is a branched polymer with a backbone of disaccharide (α-1,4-D-GalA- α-1,2-LRha) repeats in which the Rha residues can be substituted with β-1,4-galactan, branched arabinan and/or arabinogalactan side chains. The structure of RG-II is highly complex: the side chains are attached to a backbone of HG, and these complex side chains are composed of 12 types of glycosyl residues linked together by at least 22 different glycosidic bonds [2,3].

Several in vitro studies have shown that various forms of modified pectin have antitumor properties (for a review, see [4]). The RG-I region of okra pectin reduces proliferation and induces apoptosis in melanoma cells [5], and pectin oligosaccharides also induce apoptosis in myeloma cells [6]. Jackson *et al* showed that different fragmentation protocols of pectin can lead to differences in pectin apoptosis-inducing activity, and that fragmented pectin has a cytotoxic effect in androgen-dependent and -independent prostate cancer cells. Moreover, these authors showed that pH-modified citrus pectin had little or no apoptotic activity [7]. *In vivo*, it has been shown that angelan, a pectin-derived polysaccharide, could prevent melanoma cell growth and metastasis, and angelan was also reported to be an immunomodulator that enhances the immune function of B cells, macrophages and natural killer cells [8]. Oral intake of soluble pectin fragments inhibits the growth and metastasis of transplanted tumors in mice [9,10], and it has been shown that modified citrus pectin inhibits the growth of colon cancer and liver metastasis [11]. However, the mechanisms by which modified pectin exerts these effects are not known.

The purpose of this study is to characterize the cytotoxic effects of heat-fragmented citrus pectin (HFCP) on two tumor cell lines: HepG2 cells from human hepatocarcinoma and A549 cells from human lung carcinoma. We report that heat-fragmented citrus pectin induces an apoptotic-like cell death that is partly caspase-independent and activates autophagy in these two cell lines.

## Materials and Methods

### Fractionation of citrus pectin by heat treatment

Heat-fragmented citrus pectin (HFCP) was obtained according to the method described by Jackson *et al* [7]. A solution of 0.1% of citrus pectin (Sigma P9135, which is mainly composed of homopolygalacturonic acid) in double distilled water was heated for 60 min at 123°C and under a pressure of 17.2–21.7 psi. The solution was then frozen at −80°C and lyophilized. The dry material was stored at 4°C. Fresh solutions in culture medium were prepared just before being added to the cells for the incubations.

### Cell culture and pectin incubation

HepG2, A549, MCF-7 and MCF10A cells were obtained from the American Type Culture Collection HepG2 cells and MCF-7 cells were cultured in DMEM medium (Gibco 31825-023) and A549 cells in MEM medium (Gibco 41090-028). For routine culture, media were supplemented with 10% fetal bovine serum (Gibco 10270), and the cells were kept at 37°C in an atmosphere of 95% air and 5% CO_2_. MCF10A cells were cultured in DMEM/F12 medium (Gibco 11320-074) containing 5% horse serum (Gibco 16050-122), 20 μg/ml EGF, 0.5 μg/ml hydrocortisone, 10 μg/ml insulin and 100 ng/ml cholera toxin. For treatment, the cells were allowed to adhere for 24 h after seeding. The medium was removed, and the cells were washed twice with PBS (Lonza BE17-516F) and placed in a medium without serum containing the following: 3 mg/ml of filter-sterilized HFCP, 3 mg/ml of filter-sterilized citrus pectin 3 mg/ml or 50 μM etoposide, used as a positive control. Negative controls were cells incubated in medium alone. In some experiments, when indicated, staurosporine (Sigma S4400), pan-caspase inhibitor Z-VAD-fmk (BD Pharmingen 550377), 3-methyladenine (Sigma M9281) or bafilomycin (Sigma B1793) was added at the same time as etoposide, HFCP or pectin.

### Cell viability assay

HepG2 cells were seeded at 50 000 cells/well and A549 cells at 30 000 cells/well in 24-well plates before treatments for 6, 24 or 48 h. MTT (3-[4,5-dimethylthiazol-2-yl]-2,5- diphenyltetrazolium bromide, Sigma M2128) solution was prepared at a concentration of 2.5 mg/mL in phosphate-buffered saline, and 500 μL was added per well. After 2 h at 37°C and 5% CO_2_ atmosphere, the medium and MTT solution were removed before adding lysis buffer. The optical density was measured 1 h later at 570 nm using a microplate spectrophotometer (Bio-Rad x Mark Microplate spectrophotometer) and *Microplate manager 6* software.

### Cell cytotoxicity assay

HepG2 cells were seeded at 50 000 cells/well and A549 cells at 30 000 cells/well in 24-well plates before incubation for 24 or 48 h. For each well, lactate dehydrogenase activity was measured in the supernatant, in detached cells and in adherent cells after lysis in PBS containing 10% Triton X-100 (Merck 9036-19-5). The lactate dehydrogenase activity was detected by a colorimetric assay using a cytotoxicity kit (Roche 11644 793 001) and a microplate spectrophotometer. Cytotoxicity percentages were calculated by the ratio of the quantity of LDH present in the supernatant and in the detached cells of the total quantity of LDH, as in the following formula: 100* (a + b)/ (a + b + c), where a = supernatant LDH; b = detached cells LDH; c = adherent cells LDH.

### DNA fragmentation assay

HepG2 cells were seeded at 50 000 cells/well and A549 cells at 30 000 cells/well in 24-well plates before incubation for 6, 24 or 48 h. A cell death detection ELISA (Roche 11 544 675 001) was performed according to the manufacturer’s instruction. The absorbance was measured using a spectrophotometer at 405 nm. Normalization for the protein content from sibling plates determined using the Pierce reagent was performed.

### Caspase-3 activity

HepG2 and A549 cells were seeded at 600 000 cells/well in 6-well plates before treatments for 24 or 48 h. The cells were harvested on ice in PBS and centrifuged at 4°C at 1000 x g for 5 min. The cell pellets were homogenized in lysis buffer for 15 min at 4°C, and the soluble proteins were collected by centrifugation at 4°C. Samples were prepared to obtain 20 μg of proteins per 100 μL of distilled water, to which 50 μl of reaction buffer and 1 μl of Ac-DEVD-AFC substrate were added. The samples were incubated at 37°C for 60 min, and fluorescence was detected at 505 nm after excitation at 400 nm using a Fluoroscan. The assay for caspase-3 activity was performed according to Cosse *et al* [12].

### Western blot analysis

Cell lysates were prepared in lysis buffer (40 mM Tris; pH 7.5, 150 mM KCl, 1 mM EDTA, 1% Triton X-100) containing a protease inhibitor cocktail (Complete from Roche Molecular Biochemicals; 1 tablet in 2 ml of H_2_O, added at a 1: 25 dilution) and phosphatase inhibitor buffer (25 mM NaVO_3_, 250 mM PNPP, 250 mM α-glycerophosphate and 125 mM NaF, at a 1: 25 dilution) according to Cosse *et al* [12]. The medium was centrifuged, and pelleted cells were added to cell lysates. The lysates were then centrifuged at 12 000 x g for 5 min, and the supernatants were collected. The proteins (15 μg) were denatured with the addition of LDS sample buffer (Invitrogen NP0007) and heated to 70°C for 10 min. The proteins were resolved on a 4–12% NuPAGE (Invitrogen) gel and transferred to a low-fluorescence membrane (Millipore IPFL00010). The membranes were kept for 1 h in LiCor blocking solution and probed overnight with either an anti-caspase-3 rabbit antibody (Cell Signaling #9662S) that recognizes the full-length and cleaved forms of caspase-3 at a dilution of 1/500, an anti-PARP mouse antibody (BD Biosciences #551024) at a dilution of 1/1000, an anti-caspase-8 mouse antibody (Cell Signaling #97465) at a dilution of 1/1000, a mouse anti-ubiquitin antibody (LifeSensor VU101) at a dilution of 1/1000, an anti-LC3 mouse antibody (NanoTools 0260-100/LC3-2G6) at a dilution of 1/600 or an anti-p62 mouse antibody (AB Nova # H00008878-M03) at a dilution of 1/500. An anti-ß-actin mouse antibody (Sigma T5168) was used at a dilution of 1/30 000 for ß-actin immunodetection as a loading control. All antibodies were diluted in LiCor solution containing 0.1% Tween 20 (Sigma P1379). The membranes were rinsed with PBS + 0.1% Tween 20 and incubated with fluorochrome-conjugated anti-mouse or anti-rabbit antibodies (BD Bioscience #926-32221) at a dilution of 1/10 000 for 1 h in the dark at room temperature. For anti-ubiquitin labeling, the membranes were washed in PBS after transfer, fixed in PBS containing 0.5% glutaraldehyde (pH 7) and rinsed three times in PBS before blocking. The membranes were rinsed once more with PBS + 0.1% Tween 20 and dried before being scanned and analyzed using Odyssey software.

### Immunofluorescence labeling

HepG2 cells were seeded at 50 000 cells/well and A549 cells at 30 000 cells/well in 24-well plates 24 h before incubation with HFCP, pectin or etoposide for 24 h. After incubation, the cells were fixed and permeabilized for 10 min with a cold solution of 80% methanol and 20% acetone, rinsed three times with PBS-2% BSA and incubated for 2 h with primary antibodies. The primary antibody for LC3 staining was rabbit anti-LC3 (L7543 Sigma) (1/250 dilution), and the primary antibody for LAMP1 staining was mouse anti-LAMP1 (H4A3 received from August & Hildreth, Baltimore [13]). The cells were washed three times with PBS-2% BSA and then incubated for 1 h with the secondary antibodies. Alexa Fluor-488-conjugated anti-rabbit IgG antibody and Alexa Fluor-568-conjugated anti-mouse IgG antibody (Molecular Probes) were used at 1/1000 dilution. After 1 h of incubation, the cells were rinsed three times with PBS. For nuclear labeling, the cells were incubated for 30 min with TOPRO–3 (Molecular Probes, dil. 1/80) in the presence of 2 mg/ml RNAse and then rinsed three times with PBS. Finally, coverslips were mounted using Mowiol (Sigma, St Louis, USA), and the cells were observed with a fluorescent confocal microscope (Leica SP5).

## Results

### Heat-fragmented citrus pectin induces HepG2 and A549 cell death

To study the cell death-inducing effects of HFCP, two cancer cell lines, HepG2 and A549 cells, were exposed for different time periods to HFCP. The concentration of 3 mg/ml was chosen after testing several concentrations of HFCP on both cell types for 24 h (S1 Fig.). Etoposide at a concentration of 50 μM was used as a positive control. Cell morphology analyzed using phase-contrast microscopy showed that etoposide induces a slight mortality in A549 cells, whereas the HepG2 cells appeared to be more sensitive to this drug. Furthermore, cell death increased with the incubation time. HFCP at a concentration of 3 mg/ml also induced cell death in both cell types but to a much higher extent than etoposide at a concentration of 50 μM; its effect was also time-dependent. Conversely, unmodified pectin at a concentration of 3 mg/ml did not affect cell morphology (S2 Fig.).

**Fig 1:**
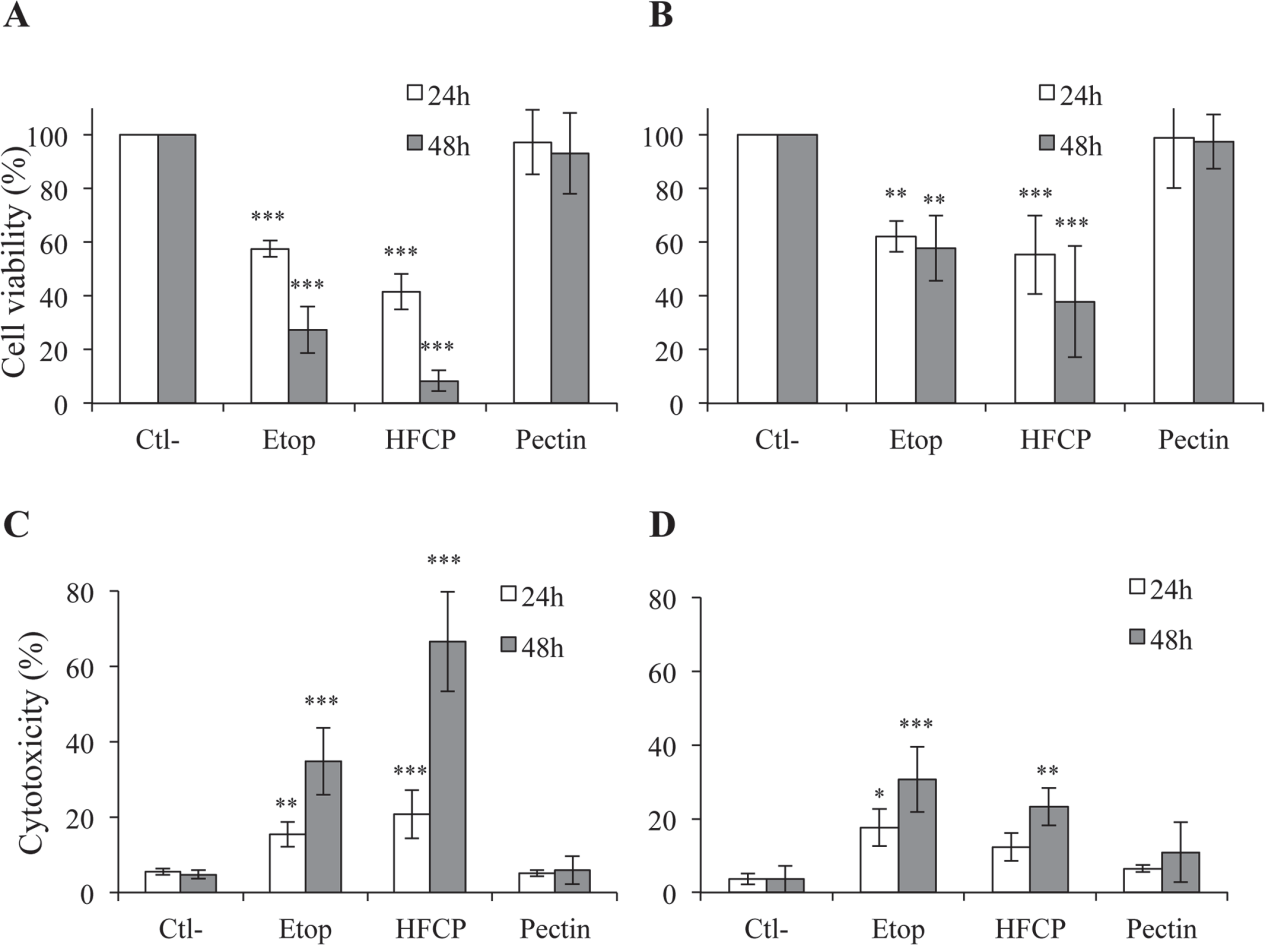
HFCP induces HepG2 and A549 cell death. HepG2 cells and A549 cells were incubated with medium alone (Ctl-), 50 μM etoposide (Etop), 3 mg/ml heat-fragmented citrus pectin (HFCP) or 3 mg/ml citrus pectin (Pectin). **(A) and (B)** Cell viability was assayed with the MTT assay in HepG2 cells (A) and A549 cells (B) after 24 and 48 h. **(C) and (D)** Cytotoxicity was assayed in HepG2 cells (C) and A549 cells (D) using an LDH cytotoxicity detection kit after 24 and 48 h of incubation. Data are the means of at least 3 replicates from independent experiments, each performed in triplicate +/−SD (n = 3–8). The statistical analyses performed were the Hartley test and ANOVAII test. P values in comparison to the corresponding control are *: P ≤ 0.05; **: P ≤ 0.01; ***: P ≤ 0.001.

MTT assays were performed after 24 and 48 h (Fig. 1A and 1B). The results showed that HFCP decreased the viability of HepG2 and A549 cells at both incubation times, whereas unmodified citrus pectin did not. HFCP toxicity increased with the incubation time and was more severe than that induced by etoposide, especially for the HepG2 cells. To confirm these data, LDH release was assayed after 24 and 48 h (Fig. 1C and 1D), and the results confirmed the cytotoxic effect of HFCP on HepG2 and A549 cells, with a stronger effect on the former.

To investigate whether serum could be protective against this HFCP-induced cytotoxicity, as observed for some other apoptosis inducers such as etoposide, HepG2 cells were incubated 24 h in the presence of HFCP in a medium supplemented with 10% fetal calf serum Staurosporine was used as the positive control since its apoptotic effect is not inhibited by serum. However, the cytotoxic effect of HFCP was not inhibited at all by serum under these conditions (S3 Fig.). Experiments have also been performed with lower concentrations for a longer incubation time (72 h) using HepG2 cells. This was performed with and without serum. As compared to 24 h of incubation, 1 mg/ml HFCP induced a more important decrease in the number of viable cells after 72 h of incubation. Serum seemed to be slightly protective after 72 h of incubation, which was not the case after 24 h of incubation (S4 Fig.).

The effect of HFCP has also been tested on normal cells. This was performed with high concentrations for a short incubation time (24 h) as well as with low concentrations for a long incubation time (72 h), with and without serum. MCF-10A cells have been used for these experiments. MCF10A cells are an immortalized, non-transformed epithelial cell line derived from human fibrocystic mammary tissue. They are often considered as a normal control for cancer cells. Results showed that HFCP also induced a strong cytotoxic effect in these cells. These results indicate that HFCP is probably not specific to cancer cells (S5 Fig.). It has to be noted that etoposide and staurosporine were also as toxic for MCF10A cells as for HepG2 cells while etoposide is presently used in clinics to treat several types of cancer.

### Fragmented citrus pectin induces non-canonical apoptosis in HepG2 and A549

**Fig 2:**
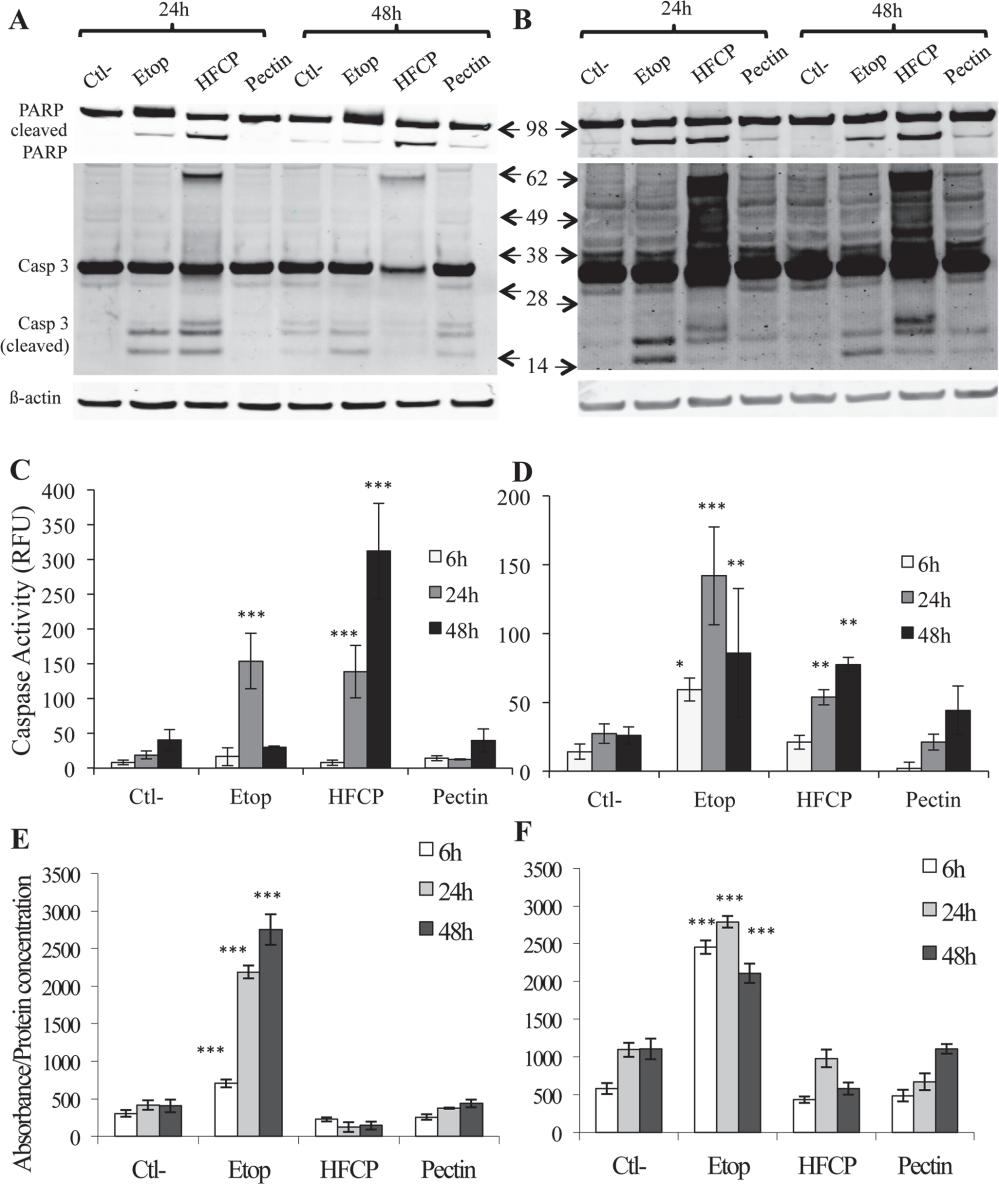
HFCP induces apoptosis in HepG2 and A549 cells. HepG2 and A549 cells were incubated with medium alone (Ctl-), 50 μM etoposide (Etop), 3 mg/ml heat-fragmented citrus pectin (HFCP) or 3 mg/ml citrus pectin (Pectin) for 24 and 48 h. **(A) and (B)** Detection of activated caspase-3 and cleaved PARP in HepG2 (A) and A549 cells (B) by western blot analysis. Immunodetection of α-tubulin was used as the loading control. The results are representative of three independent experiments. **(C) and (D)** Caspase-3 activity was assayed using a specific fluorogenic substrate after 6, 24 and 48 h of incubation in HepG2 cells (C) and A549 cells (D). **(E) and (F)** DNA fragmentation was assayed using an ELISA detection kit after 6, 24 and 48 h of incubation in HepG2 cells (E) and in A549 cells (F). Data are the means of triplicates +/−SD (n = 3). Data are the means of triplicates +/−SD (n = 3). The statistical analyses performed were the Hartley test and ANOVAII test. P values in comparison to the corresponding control are *: P ≤ 0.05; **: P ≤ 0.01; ***: P ≤ 0.001.

To investigate whether pectin fragments induce apoptosis, we analyzed the activation of caspase-3 by a western blot analysis. The activation of caspase-3 occurred through cleavage, resulting in fragments of 14 kDa and 17 kDa, as observed when the cells were incubated with etoposide for 24 or 48 h (Fig. 2A). HepG2 cells incubated for 24 or 48 h with HFCP displayed different forms of cleaved caspase-3 than the cells incubated with etoposide. At 24 h, additional bands of higher apparent molecular weights, approximately 20 and 60 kDa, appeared; at 48 h, the cleaved fragments of caspase-3 were less abundant in the cells incubated with HFCP, but the 60-kDa form of caspase-3 was still present (Fig. 2A). After 48 h of incubation, a fragment of 20 kDa appeared in the cells exposed to medium alone, etoposide and unfragmented pectin, most likely because these cells were incubated without serum. Regardless, the 60-kDa band was specific to the HFCP-incubated cells.

Caspase cleavage and additional bands were also noticeable in A549 cells after 24 or 48 h of HFCP treatment (Fig. 2B). Moreover, the caspase-3 cleavage pattern in A549 cells incubated with HFCP displayed an intriguing smear from 30 to 60 kDa (Fig. 2B). A western blot analysis for the PARP protein (Fig. 2A and 2B), a substrate of caspase-3, showed the cleavage of this protein in both cell types incubated with HFCP or etoposide for both time periods. PARP cleavage was also detected in HepG2 cells incubated for 48 h with medium alone or unmodified pectin, though cleaved PARP was less abundant in these cells than in the HFCP-incubated cells. A549 cells incubated for 24 or 48 h with unmodified pectin showed a barely detectable amount of cleaved PARP, most likely because the incubation was in serum-free medium.

To determine whether the non-conventional cleavage of caspase-3 led to enzyme activation, caspase-3 activity was assayed in HepG2 and A549 cells incubated with 50 μM etoposide, 3 mg/ml HFCP or 3 mg/ml pectin for 8, 24 and 48 h (Fig. 2C and 2D). The results showed that, after 24 h of incubation, etoposide strongly increased caspase-3 activity in HepG2 cells when compared to control cells. The HepG2 cells treated with HFCP also showed a higher caspase-3 activity that increased with incubation time. The A549 cells treated with etoposide showed a small increase in caspase-3 activity when compared to the control cells, though this increase in caspase-3 activity was less important than that observed in HepG2 cells but was nonetheless statistically significant. HFCP-exposed A549 cells also displayed caspase-3 activation that increased over time.

To confirm that HFCP induces apoptosis, DNA fragmentation, a late characteristic of apoptosis, was assessed. A free nucleosome ELISA kit was used to measure the release of free nucleosomes from DNA (Fig. 2E and F), and the results revealed that HepG2 and A549 cells treated with etoposide showed DNA fragmentation leading to nucleosome release. In the contrast to what was observed for etoposide, DNA fragmentation was not observed in cells incubated with HFCP, indicating that HepG2 and A549 cells treated with fragmented pectin do not show classical apoptotic features. This is consistent with the nuclear morphology observed after DAPI staining, which did not display typical apoptotic fragmented features but appeared to be shrunken.

A viability assay was also performed in caspase-3-deficient MCF7 cells (S6 Fig.). The results showed a decrease in viability of these cells when incubated in the presence of HFCP for 24 or 48 h, suggesting that caspase-3 activation is not needed for inducing cell death in response to HFCP exposure.

### Role of caspases in HFCP-induced cell death

**Fig 3:**
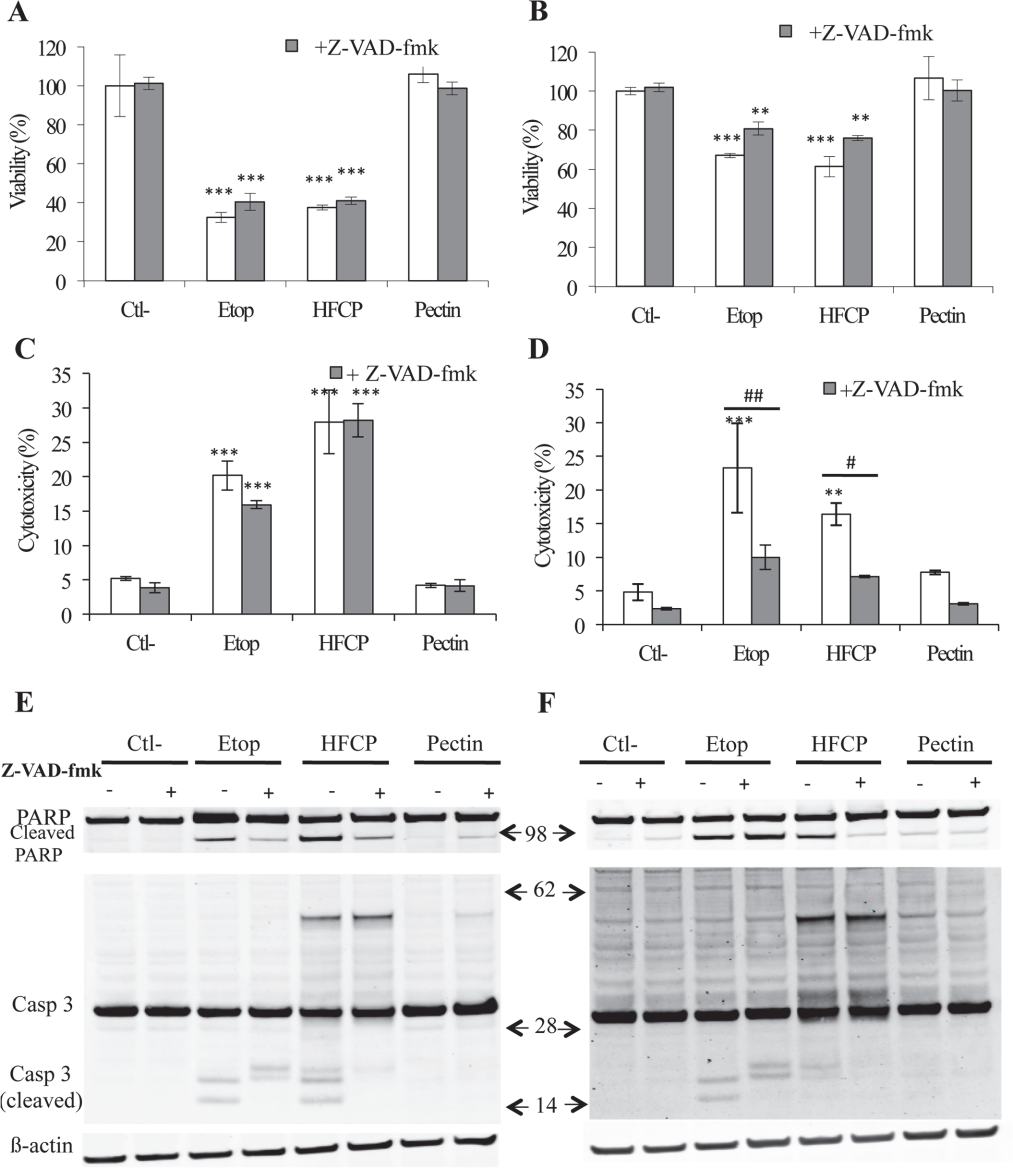
HFCP-induced apoptosis is partially inhibited by caspase inhibition. HepG2 and A549 cells were incubated with medium alone (Ctl-), 50 μM etoposide (Etop), 3 mg/ml heat-fragmented citrus pectin (HFCP) or 3 mg/ml citrus pectin (Pectin) in the presence or absence of Z-VAD-fmk, a caspase inhibitor, at 20 μM. **(A) and (B)** Cell viability of HepG2 cells (A) and A549 cells (B) was measured with the MTT assay after 24 h of incubation. **(C) and (D)** Cytotoxicity was assayed in HepG2 (C) and in A549 (D) after 24 h of incubation using an LDH cytotoxicity detection kit. Data are the means of triplicates +/−SD (n = 3). The statistical analyses performed were the Hartley test and ANOVAII test. P values in comparison to the corresponding control are *: P ≤ 0.05; **: P ≤ 0.01; ***: P ≤ 0.001. **(E) and (F)** A western blot analysis of activated caspase-3 and cleaved PARP was performed on 15 μg of protein from HepG2 cells (E) and A549 cells (F) after 24 h of incubation. Immunodetection of ß-actin was used as the loading control. The results are representative of two independent experiments.

To investigate whether caspases play a major role in the cell death induced by HFCP, cells were incubated with HFCP in the presence of a pan-caspase inhibitor (Z-VAD-fmk), and the viability of and cytotoxicity to HepG2 and A549 cells after 24 h were analyzed. The caspase inhibitor led to a small increase in viability in both cell types incubated with etoposide (Fig. 3A and 3B), a small decrease in cytotoxicity in HepG2 cells incubated with etoposide and a marked decrease in cytotoxicity in A549 cells incubated with etoposide (Fig. 3C and 3D). This set of data suggests that Z-VAD-fmk was able to prevent, at least in part, the cell death associated with caspase activity. However, HepG2 cells incubated with HFCP in the presence of Z-VAD-fmk did not show any increase in viability when compared to cells incubated with HFCP alone. A cytotoxicity assay confirmed these observations (Fig. 3A and 3C), thus indicating that caspases did not contribute to the HepG2 cell death induced by HFCP. However, A549 cells incubated with HFCP and caspase inhibitor showed a higher viability and much less cytotoxicity than the A459 cells incubated with HFCP alone, suggesting that caspases could be involved in the death induced by HFCP in A549 cells (Fig. 3B and 3D).

In an attempt to understand the differential effect of the caspase inhibitor on these two cell types, we verified that Z-VAD-fmk did inhibit caspase activity at the concentration used in this work (S1 Table). Indeed, the results showed that Z-VAD-fmk did completely inhibit etoposide- and HFCP-induced caspase activation. We next analyzed caspase-3 cleavage and PARP cleavage in the absence or in the presence of Z-VAD-fmk by western blotting (Fig. 3E and 3F). The fragmentation of caspase-3 that was observed when cells were exposed to etoposide was no longer observed when Z-VAD-fmk was added, a result that is in concordance with the literature [14]. The non-canonical fragmentation of caspase-3 that was observed when HepG2 and A549 cells were treated with HFCP was also inhibited, and the pattern of caspase-3 cleavage became comparable to that observed for cells exposed to etoposide in the presence of Z-VAD-fmk, except for the 60-kDa band that was still detected in both cell types incubated with HFCP and Z-VAD-fmk.

These data raise the possibility that the “non-conventional” caspase-3 cleavage we observed could be due to other caspases or proteases and thus might not be related to classical apoptosis. Indeed, HFCP-induced PARP protein cleavage was only partially inhibited by Z-VAD-fmk, indicating that there might be mechanism other than caspase activation that could be involved. In addition, the PARP protein cleavage induced by etoposide was not inhibited by Z-VAD-fmk in A549 cells, whereas the PARP protein cleavage induced by HFCP treatment was completely inhibited.

**Fig 4:**
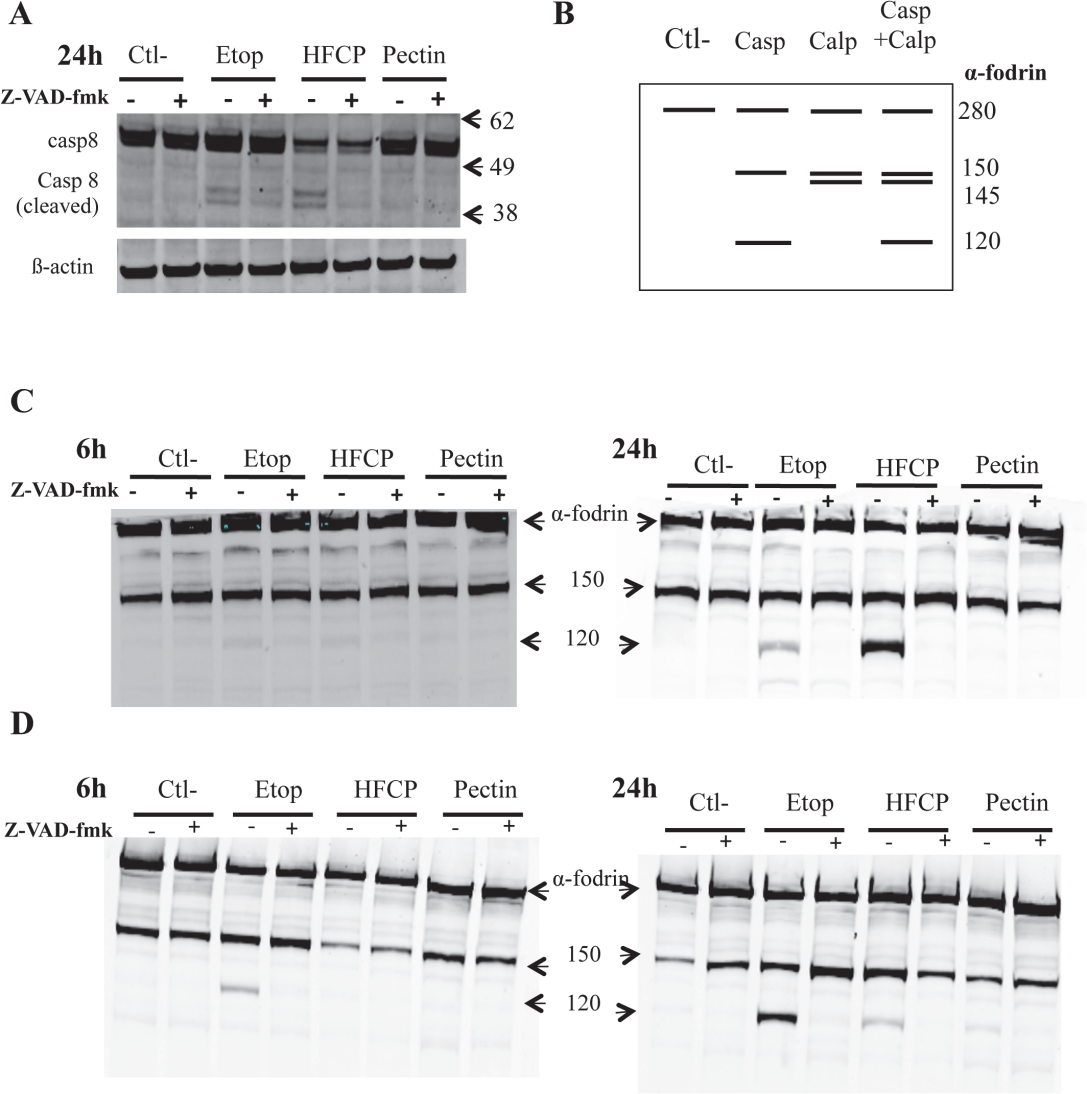
HFCP induces caspase-8 and α-fodrin cleavage. HepG2 cells were incubated with medium alone (Ctl-), 50 μM etoposide (Etop), 3 mg/ml heat-fragmented citrus pectin (HFCP) or 3 mg/ml citrus pectin (Pectin) **(A)** Z-VAD-fmk at 20 μM was added or not to the cells during incubation. The detection of activated caspase-8 in HepG2 cells by western blot analysis was performed on 15 μg of protein after 24 h of incubation. The immunodetection of ß-actin was used as the loading control. **(B)** Theoretical illustration of α-fodrin cleavage resulting from the activation of caspase (casp), calpain (calp) or the combination of caspase and calpain (casp+calp). **(C)** and **(D)** HepG2 and A549 cells were incubated with medium alone (Ctl-), 50 μM etoposide (Etop), 3 mg/ml heat-fragmented citrus pectin (HFCP) or 3 mg/ml citrus pectin (Pectin), and 20 μM Z-VAD-fmk was added or not to the cells for 6 or 24 h of incubation. The detection of α-fodrin in HepG2 **(C)** and A549 **(D)** cells by western blot analysis was performed on 15 μg of protein.

Because effector caspases are activated by initiator caspases, caspase-8 cleavage was analyzed in HepG2 cells by a western blot analysis (Fig. 4A). The result showed that the incubation of HepG2 cells in the presence of etoposide or HFCP did generate cleaved fragments of caspase-8 with a molecular weight of 43 and 41 kDa (Fig. 4A). The cleavage of caspase-8 in cells exposed to etoposide is consistent with the literature [15], and this cleavage was prevented by the presence of Z-VAD-fmk in both etoposide- and HFCP-exposed cells (Fig. 4A). Moreover, the abundance of the full-length procaspase decreased in the HepG2 cells incubated with HFCP and was not prevented by the addition of Z-VAD-fmk. Hence, the decrease in abundance of procaspase-8 was not due to cleavage caused by other caspases because Z-VAD-fmk did not prevent it.

Calpains are proteases that are also activated in response to stress and may participate in cell death. As the study of the α-fodrin cleavage pattern could inform on caspase or calpain activation [16,17], the fragmentation of α-fodrin was assessed by a western blot analysis in HepG2 and A549 cells after 6 and 24 h of incubation with or with Z-VAD-fmk addition to determine whether calpains were activated during HFCP-induced cell death (Fig. 4 C, D). The theoretical cleavage pattern of α-fodrin is shown in Fig. 4B. Full-length α-fodrin has a molecular weight of 280 kDa; α-fodrin cleaved by caspase shows bands of 150 and 120 kDa, and α-fodrin cleaved by calpain shows bands of 150 and 145 kDa. In both cell types exposed to etoposide or HFCP (Fig. 4C and D), the full-length form of the protein was detected, as was a fragment of 150 kDa. This fragment could represent the cleavage of α-fodrin at a hypersensitive site by low levels of endogenously active proteases [18]. After 24 h of incubation, the α-fodrin cleavage pattern in HFCP-incubated cells was similar to the cleavage pattern in etoposide-incubated cells, and both presented a fragment of 120 kDa. As the 120-kDa α-fodrin product is associated with caspase-3 activation [19] and the formation of this fragment was inhibited when Z-VAD-fmk was added in both cases, the results indicate that HFCP induced the activation of some caspases in both cell types.

**Fig 5:**
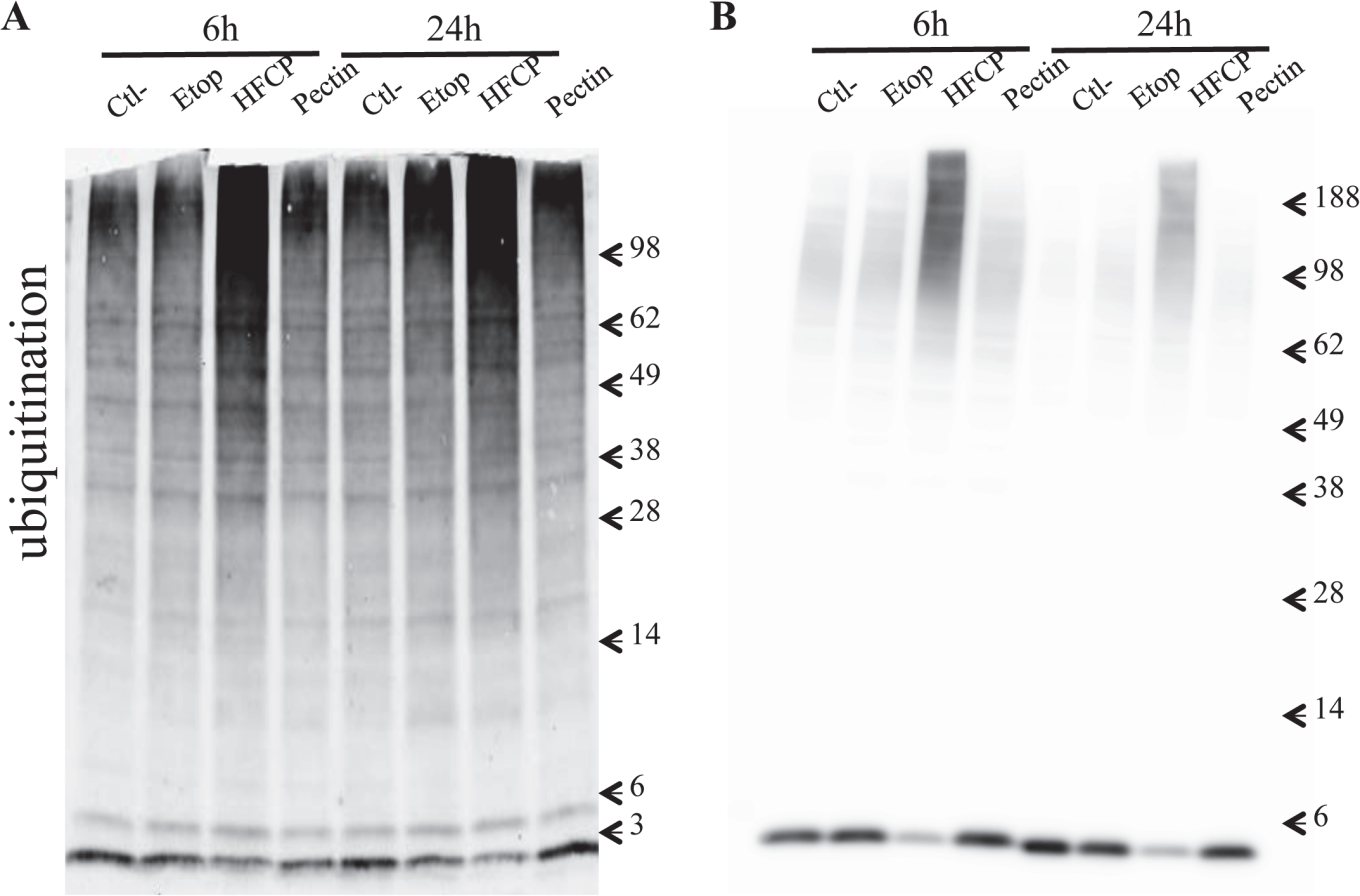
HFCP induces protein ubiquitination. HepG2 cells **(A)** and A549 cells **(B)** were incubated with medium alone (Ctl-), 50 μM etoposide (Etop), 3 mg/ml heat-fragmented citrus pectin (HFCP) or 3 mg/ml citrus pectin (Pectin) for 6 and 24 h. The detection of ubiquitylated proteins by western blot analysis was performed on 15 μg of protein.

Caspase 8 activation has been shown to be induced in a receptor-independent manner due to proteasome inhibition [20]. Because no receptor has yet been reported for HFCP and to investigate whether HFCP could activate such a pathway, protein ubiquitination was assessed by a western blot analysis in HepG2 and in A549 cells. An early increase in ubiquitination was observed when the cells were incubated in the presence of HFCP for 6 h but not in the presence of medium alone, etoposide or unmodified pectin which was still detectable after 24 h of incubation (Fig. 5A and B).

### HFCP incubation induces autophagy activation in HepG2 and A549 cells

Apoptosis is no longer considered as the only type of programmed cell death (PCD), and autophagy is now accepted as another type of PCD [21].

Because our data suggest that apoptosis might not, or only partially, be the mechanism responsible for HFCP-induced cell death, we investigated whether HFCP could also induce autophagy by western blotting analyses for LC3 and p62 proteins. An increase in LC3II abundance and p62 degradation are usually correlated with the activation of autophagy [22].

**Fig 6:**
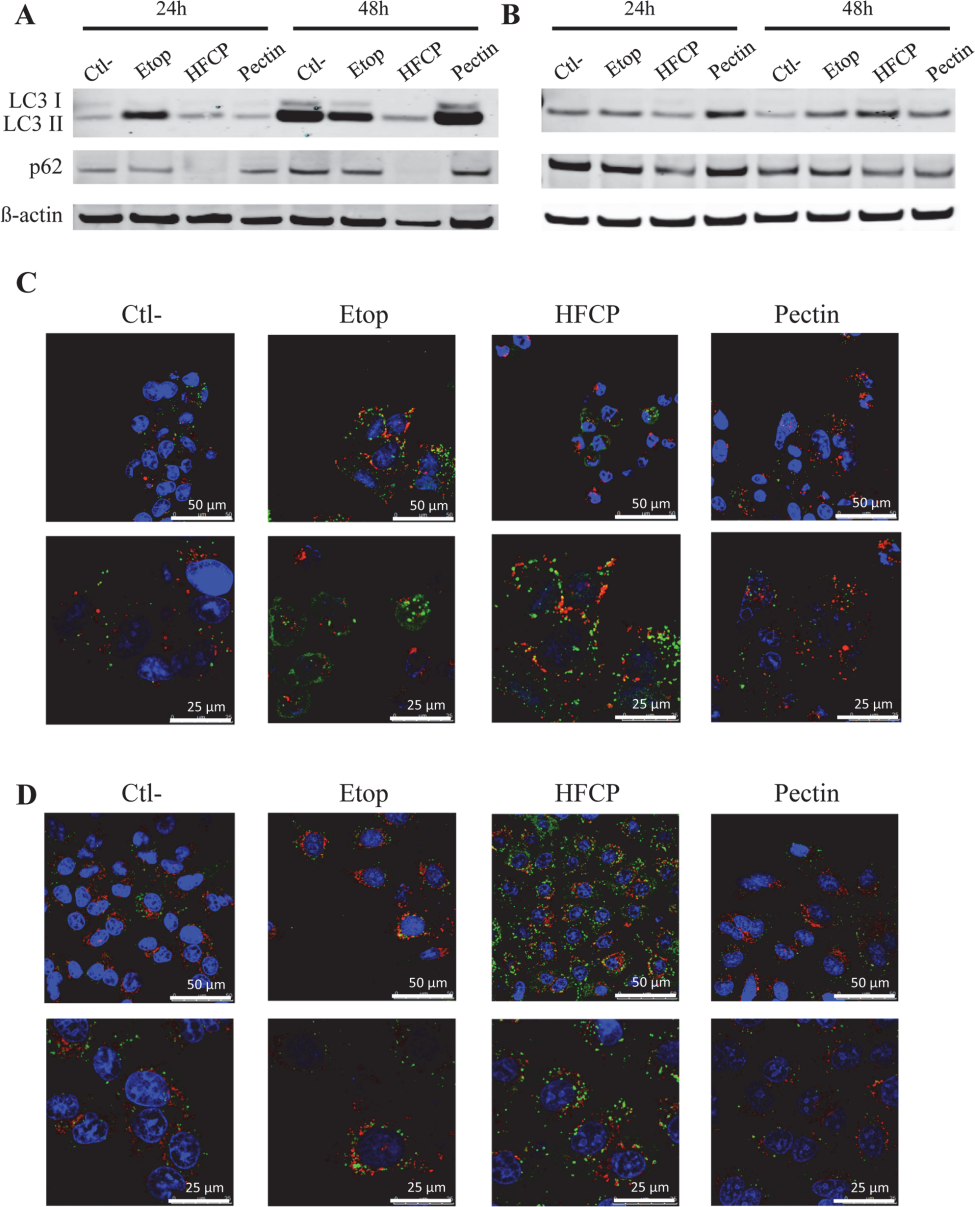
HFCP induces autophagy in HepG2 and A549 cells. HepG2 and A549 cells were incubated with medium alone (Ctl-), 50 μM etoposide (Etop), 3 mg/ml heat-fragmented citrus pectin (HFCP) or 3 mg/ml citrus pectin (Pectin). **(A, B)** A western blot analysis was performed on 15 μg of protein from HepG2 **(A)** or A549 **(B)** cells using antibodies against LC3 and p62. The immunodetection of ß-actin was used as a load control. The results are representative of two independent experiments. **(C, D)** Immunolabeling of LC-3 (green) and LAMP1 (red) in HepG2 **(C)** or A549 **(D)** cells incubated for 24 h. Nuclei were stained with TO-PRO-3 (blue), and cells were observed using a confocal microscope with the photomultiplier remaining constant. Pictures taken at two magnifications are shown.

HepG2 cells incubated with HFCP for 24 h showed a slight conversion of LC3I to LC3II; however, this effect was smaller than that induced by etoposide (Fig. 6A). In addition, the abundance of p62 decreased when HepG2 cells were exposed to HFCP but not to etoposide (Fig. 6A). After 48 h of incubation, LC3II also accumulated in the control cells, most likely because the cells were incubated without serum. This effect was less pronounced in the presence of etoposide or HFCP.

The pattern of LC3 was also assessed by immunofluorescence labeling. A punctuated pattern of LC3 was observed in HepG2 cells incubated with etoposide or HFCP (Fig. 6C). However, only minimal co-localization of LC3 from autophagosomes and LAMP1 from lysosomes was observed, both in cells incubated with etoposide and in cells incubated with HFCP.

After 24 h of incubation, the abundance of LC3II increased in etoposide and pectin-incubated A549 cells but not in HFCP-incubated cells (Fig. 6B), whereas the abundance of p62 did decrease in HFCP-incubated cells. In addition, an incubation of 48 h led to the accumulation of LC3II in the etoposide- and HFCP-treated cells. Surprisingly, the LC3I form was not detectable by western blotting for A549 cells, and the abundance of the p62 protein decreased in HFCP-incubated A549 cells after 24 and 48 h. However, the induction of autophagy appears to be less important in A549 cells compared to HepG2 cells. The pattern of LC3 staining observed by confocal microscopy was clearly highly punctuated in A549 cells incubated with HFCP for 24 h (Fig. 6D).

**Fig 7:**
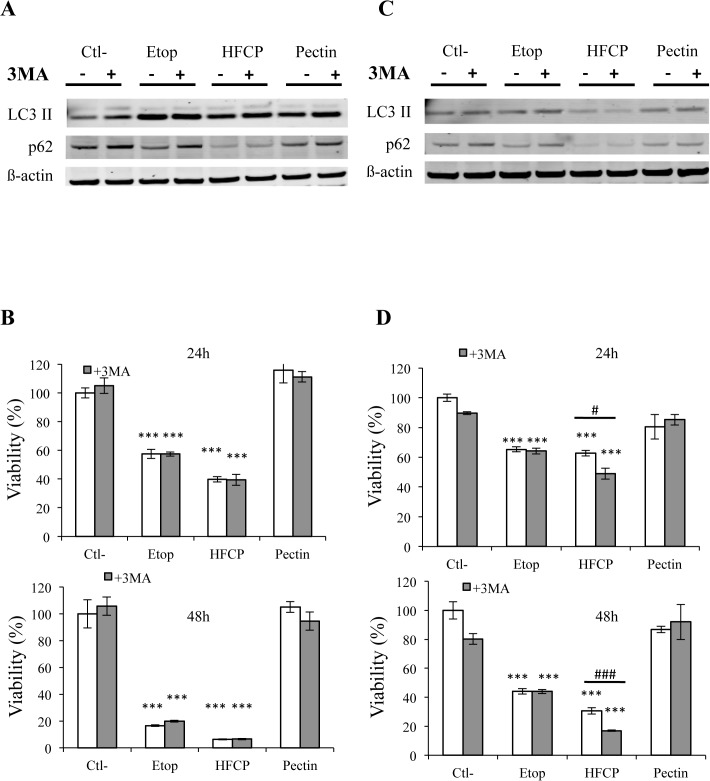
Inhibition of autophagy using 3-MA. HepG2 **(A, B)** and A549 **(C, D)** cells were incubated with medium alone (Ctl-), 50 μM etoposide (Etop), 3 mg/ml heat-fragmented citrus pectin (HFCP) or 3 mg/ml citrus pectin (Pectin). 3-MA at 1 mM was added or not to cells during incubation. **(A, C)** After 24 h of incubation, a western blot analysis was performed on 15 μg of protein using antibodies against LC3 and p62. The immunodetection of ß-actin was used as the loading control. The results are representative of two independent experiments. **(B, D)** Viability was assayed using the MTT test after 24 or 48 h. Data are the means of triplicates +/−SD (n = 3). *^/#^ or ***^/###^: P ≤ 0.05 or P ≤ 0.001 using ANOVA I and Tukey’s multiple comparison test.

To examine apoptosis, we used autophagy inhibitors to assess whether autophagy could be involved in the cell death induced by HFCP. 3-Methyladenine (3-MA) was used to block autophagosome formation by inhibiting type III phosphatidylinositol 3-kinase, and bafilomycin was used to prevent maturation of the autophagic vacuole by inhibiting the fusion of the autophagosome with the lysosome [22]. Indeed, bafilomycin is an inhibitor of the vacuolar-type H+-ATPase (V-ATPase), thus preventing the acidification of organelles containing this enzyme, such as lysosomes and endosomes. LC3II conversion and p62 abundance in HepG2 and A549 cells after incubation in the presence of 1 mM 3-MA were assessed by a western blot analysis, and effect of these inhibitors on the cell death induced by HFCP was assessed by the MTT test. HepG2 and A549 cells incubated with 3-MA showed a small increase in the abundance of p62 in the control cells and cells exposed to etoposide, suggesting that 3-MA partly inhibited autophagy at the concentration used. However, no effect of 3-MA on p62 degradation in HFCP-incubated cells was observed (Fig. 7A, C). Incubation with 3-MA had no effect on LC3II conversion in HepG2 cells incubated with etoposide but slightly increased the abundance of LC3II in control cells and in pectin-incubated cells. With regard to A549 cells, no effect of 3-MA on LC3II abundance was observed, though a slight increase in the p62 level was observed in both the control and etoposide-exposed cells.

HepG2 and A549 cell viability was assessed after 24 and 48 h of incubation with or without 1 mM 3-MA (Fig. 7B). 3-MA did not affect the decrease in viability induced by HFCP in HepG2 cells after 24 h or 48 h. In A549 cells, 3-MA incubation slightly increased the loss of viability induced by HFCP (Fig. 7D), indicating that HepG2 and A549 do not react in a different way when exposed to HFCP and that autophagy could protect A549 cells incubated in the presence of heat-fragmented pectin.

**Fig 8:**
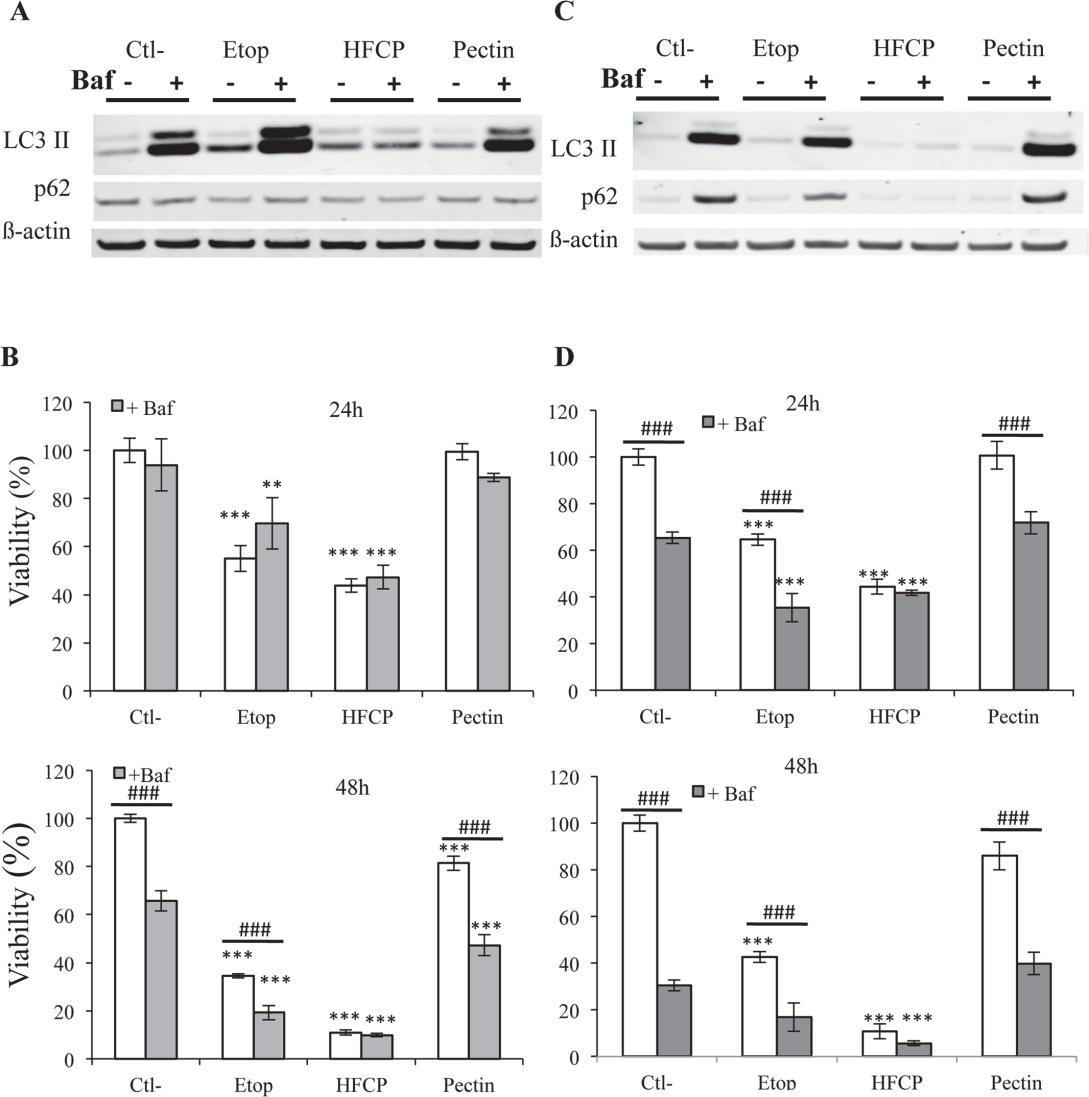
Inhibition of autophagy using bafilomycin. HepG2 **(A, B)** and A549 **(C, D)** cells were incubated with medium alone (Ctl-), 50 μM etoposide (Etop), 3 mg/ml heat-fragmented citrus pectin (HFCP) or 3 mg/ml citrus pectin (Pectin). Bafilomycin at 10 nM was added or not to cells during incubation. **(A, C)** After 24 h of incubation, a western blot analysis was performed on 15 μg of protein using antibodies against LC3 and p62. The immunodetection of ß-actin was used as the loading control. The results are representative of two independent experiments. **(B, D)** Viability was assayed using the MTT test after 24 and 48 h. Data are the means of triplicates +/−SD (n = 3). ** or ***^/###^: P ≤ 0.01 or P ≤ 0.001 using ANOVA I and Tukey’s multiple comparison test.

When the cells were incubated in the presence of 100 nM bafilomycin for 24 h, LC3II degradation was prevented; indeed, an accumulation of this protein was observed in control cells as well as in etoposide- and unmodified pectin-incubated cells. However, no accumulation of LC3II was observed in any of the cell lines incubated with HFCP (Fig. 8A, 8C). In addition, p62 degradation resulting from HFCP exposure was not inhibited, thus indicating that p62 degradation could be independent of autophagosome fusion with lysosomes.

Incubation with 100 nM bafilomycin impaired the viability of all the cells except for those incubated with HFCP, in agreement with the western blot analysis for LC3II. Thus, the HFCP-exposed cells could be insensitive to bafilomycin (Fig. 8B, 8D).

## Discussion

The anticancer activity of modified forms of pectin has previously been reported for various cell lines. Jackson *et al* studied the effect of different forms of pectin on LNCaP prostate cancer cells [7], and Yan & Katz showed that PectaSol-C Modified Citrus Pectin induced apoptosis in mouse androgen-dependent and -independent prostate cancer cells, with the cleavage of caspase-3 [23]. It has also been reported that the incubation of B16F10 melanoma cells with okra RG-I reduced their proliferation and induced apoptosis [5]. In addition, GCS-100, a polysaccharide derived from citrus pectin, induced apoptosis in multiple myeloma cells via the release of cytochrome c and the activation of caspase-3 [6]. However, the underlying mechanisms involved in the apoptotic activity of modified pectin have remained unclear and not well defined.

In this study, HepG2 and A549 cells incubated with HFCP showed evidence of apoptosis. PARP cleavage into the 89-kDa fragment is associated with apoptosis induction [24] as well as caspase-3 and -7 activation. Although PARP can be cleaved by proteins other than caspases, such proteases as calpains or cathepsins give rise to a 40-kDa fragment or 55- and 42-kDa fragments, respectively [25,26]. Thus, it is unlikely that calpains or cathepsins were activated in the cells incubated with HFCP because only an 89-kDa PARP fragment was observed. Moreover, the α-fodrin cleavage pattern suggests that caspases and not calpains are responsible for its cleavage in the etoposide- and HFCP-exposed cells of both cell types. An examination of caspase-3 fragmentation revealed the presence of fragments corresponding to the activation of caspase-3 in cells incubated with HFCP, even though unknown additional bands appeared.

However, not all the typical features of apoptosis were detected. Indeed, the HepG2 and A549 cells did not show DNA fragmentation after incubation with HFCP but displayed shrunken nuclei. Utilization of the pan-caspase inhibitor Z-VAD-fmk in HepG2 cells did not prevent HFCP-induced cell death but prevented caspase-3 cleavage and partly prevented PARP cleavage, confirming the implication of some caspases. A possible explanation for these apparent discrepancies could be that caspase-3 was inhibited, and another protease sharing some caspase-3 substrates was activated. Peltenburg’s team showed that in melanoma cells incubated with etoposide, the addition of Z-VAD-fmk is unable to prevent the cleavage of PARP despite DEVDase activity inhibition. Additionally, PARP degradation and caspase activation were both inhibited in cells pretreated with 4-(2-aminoethyl) benzensulfonyl fluoride (AEBSF), a serine protease inhibitor. These authors concluded that a serine protease regulates an alternative initiation mechanism that leads to caspase activation and PARP cleavage despite the presence of Z-VAD-fmk [27]. Such a mechanism could explain the paradoxical data we obtained and could also explain the cleavage of PARP observed in A549 cells incubated for 48 h in the presence of etoposide and Z-VAD-fmk.

The western blot analyses of caspase-3 showed a non-canonical pattern of caspase-3 cleavage and the appearance of bands with higher molecular weights than expected (mainly at 20 kDa and 60 kDa and a smear, mostly in A549 cells, between 30 and 60 kDa). This change in electrophoretic mobility was not due to phosphorylation because α-phosphatase treatment of the sample did not affect it (data not shown). Susuki *et al* proposed that active caspase-3 could be degraded by the proteasome after ubiquitination by XIAP [28], and a study by Ares *et al* proposed that the incubation of HMEC-1 cells with oxLDL results in the polyubiquitination of caspase-3 and inactivation of this protease. In HepG2 and A549 cells incubated in the presence of HFCP, we found an increase in protein ubiquitination. Ubiquitination of caspase-3 could explain the unusual migration profile of this protein in our western blot analysis. A possible explanation for the caspase-3 activity detected in HFCP-treated cells is that active caspase-3 and modified caspase-3 co-exist during this incubation time. The western blot pattern could thus represent a superposition of the patterns for two populations of caspase-3.

The inhibition of caspases with Z-VAD-fmk in HepG2 and A549 cells prevented PARP and caspase cleavage, indicating that even if these cleavages are non-canonical, they might depend on some caspases. Because caspase-8 activation was observed, this protease may be involved. It has been shown that caspase-8 could be activated through autophagy after proteasome inhibition [29]. In this situation, the caspase-8 activity is low and persists a long time. Caspase-8 possesses DEVDase activity [30], and the PARP protein is cleaved at a DEVD site [31]. However, caspase inhibition did not influence the HFCP cytotoxicity induced in HepG2 cells and reduced it slightly in A549 cells at a late time point, indicating that caspases are not a requisite for HFCP-induced cell death. These results confirm that HFCP did not induce classical apoptosis but rather a form of cell death that does not require caspase-3 activation. In addition, the incubation of MCF7 cells, which are deficient for caspase-3, with HCFP showed a decrease in viability, confirming that caspase-3 is not essential for the onset of the cell death induced by HFCP.

Autophagy is thought to have a dual role in cell death (promoting or protective) and is now accepted as a second type of programmed cell death in some circumstances [32,33]. Markers of autophagy could be detected in both cell types incubated in the presence of HFCP.

The decrease in p62 abundance observed in our western blot analysis and the marked punctuated pattern observed for LC3 staining suggest that HFCP induced the early activation of autophagy. To our knowledge, this is the first time that the activation of autophagy is shown in cancer cells treated with modified pectin. In HepG2 cells incubated in the presence of HFCP for 24 h, an increase in LC3II abundance was noted when compared to control cells. This indicates an increase in autophagosome formation. After 48 h of incubation without serum, LC3II abundance increased in HepG2 cells incubated under different conditions, except in HFCP-incubated cells. This result could be due to the inhibition of autophagy or to a higher rate of autophagic degradation in HFCP cells than in control cells [34]. Incubating HepG2 cells with 3-MA did not markedly modify LC3II abundance or influence cell viability. However, we cannot completely rule out that the 3-MA concentration used (1 mM) was too low. As the recommended concentration of 5 mM was toxic for HepG2 cells under our experimental conditions, a higher concentration of 3-MA would have had a more important impact on the HFCP-incubated HepG2 cells. Unlike 3-MA, bafilomycin had marked effect on LC3II conversion in HepG2 cells but is also toxic. Bafilomycin is an inhibitor of the vacuolar H^+^-ATPase (V-ATPase), which is indispensable for the fusion between autophagosomes and lysosomes and the subsequent maturation of autophagosomes [35]. In cells in which autophagy is impaired by bafilomycin, LC3II associates with the inner membrane of autophagosome and is no longer degraded; thus, an increase in the abundance of LC 3II can be observed [34]. This was found in the HepG2 control cells and HepG2 cells incubated in the presence of etoposide or unmodified pectin. Indeed, the abundance of LC3II increased in the presence of bafilomycin, indicating that autophagy occurred in these cells, and this induction of autophagy is consistent with a 24-h incubation period without serum. Surprisingly, HepG2 cells incubated in the presence of HFCP did not show an increase in LC3II when bafilomycin was added. These results could suggest that autophagy flow was blocked in the HFCP-exposed HepG2 cells or that bafilomycin was not efficient in these treated cells. The p62 protein is known to be an adaptor protein in selective autophagy, interacting with the ubiquitin signal on protein aggregates and as an autophagy component with LC3 via the LIR motif, allowing the formation of autophagosome to engulf the aggregates (reviewed in [36]). Impaired autophagy should not result in p62 protein degradation. In addition, p62 is also known as a shuttling factor for the delivery of polyubiquitinated substrates to proteasomes, and a depletion in p62 levels thus results in an accumulation of ubiquitinated proteins [37]. The decrease in p62 abundance observed in the HFCP-incubated HepG2 cells could explain the augmentation of ubiquitination we observed in these treated cells.

LC3II abundance increased in A549 cells incubated in the presence of HFCP only after 48 h of incubation, despite a decrease in p62 abundance and LC3II punctuation. It is important to note that the amount of LC3II can fluctuate greatly over time and that the abundance of p62 could decrease even if there is no accumulation of LC3II abundance [34]. 3-MA decreased the viability of HFCP-incubated A549 cells, suggesting that the autophagy induced by HFCP could have a protective role in A549 cells. The incubation of A549 cells with bafilomycin decreased viability except in HFCP-incubated cells at 24 and 48 h, and western blotting did not show an increase in LC3II abundance, suggesting that autophagy was blocked in HFCP-exposed A549 cells or that bafilomycin was not effective in HFCP-incubated cells, such as HepG2 cells.

Taken together, these results suggest several possible hypotheses to explain how HFCP induces cell death. The first hypothesis would be a classical apoptosis pathway. HFCP molecules could bind to a death receptor on the cell surface, inducing activation of the initiator caspase-8. Caspase-8, in turn, would then cleave and activate effector caspase-3, which then cleaves its substrates, including PARP and α-fodrin. At the same time, autophagy is induced either directly by HFCP molecules or resulting from apoptosis. In A549 cells, this autophagy could be partially protective. However, this model is too simple to explain the absence of DNA fragmentation and cannot explain the augmentation of ubiquitination observed in HFCP-treated cells.

**Fig 9:**
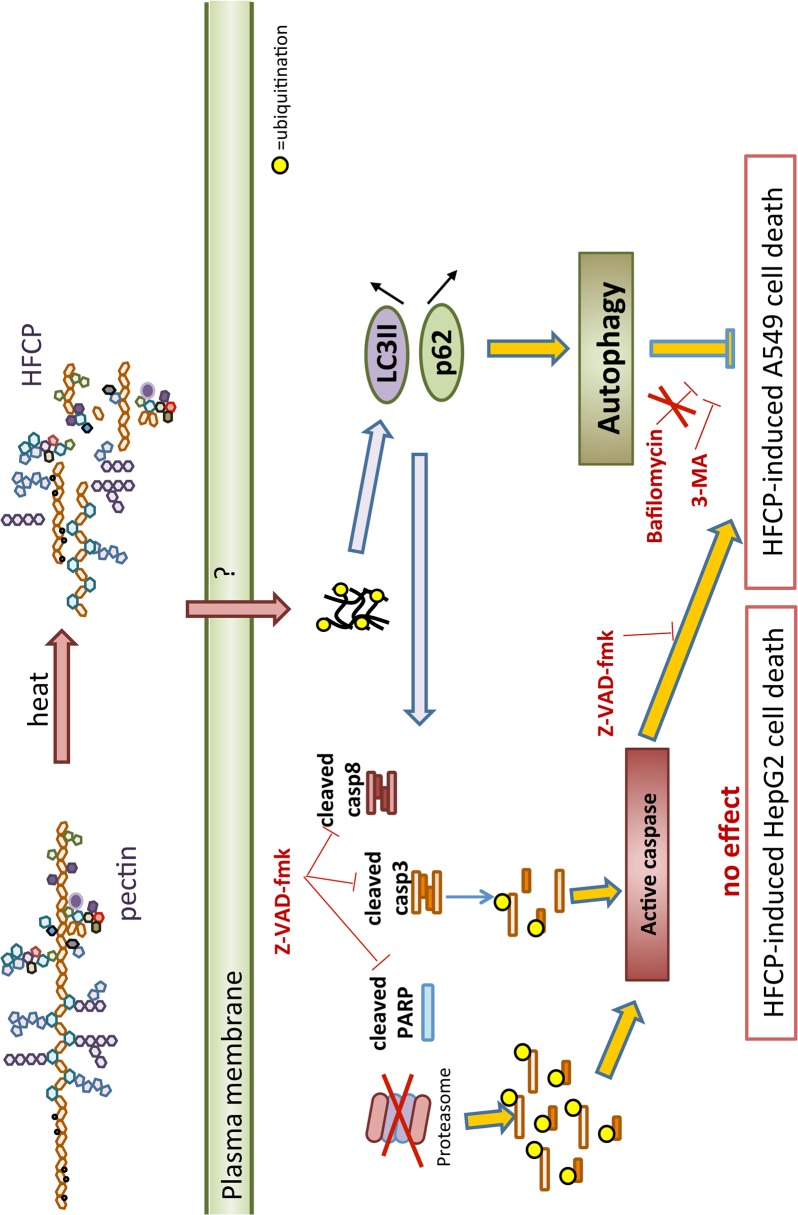
Schematic representation of possible HFCP-induced cell death mechanisms in HepG2 and A549 cells. Proteins from cells exposed to HFCP undergo ubiquitination, leading to their aggregation. The formation of p62-caspase-8 aggregates would lead to caspase-8 cleavage and its activation. Active caspase-8 induces PARP and ubiquitinates cleaved active caspase-3. The accumulation of protein aggregates also triggers autophagy, which could also trigger caspase-8 activation. However, none of these pathways appears to play a role in inducing HepG2 cell death. In contrast, Z-VAD-fmk is partly protective toward HFCP-induced cell death in A549 cells, indicating that apoptosis may participate in cell death. Furthermore, 3-MA further increases cell death, indicating that autophagy may be protective. Other alternative yet unknown mechanisms may also be involved under these conditions.

A second hypothesis could be that the incubation of cells with HFCP induced an accumulation of misfolded proteins, thus leading to their ubiquitination. Alternatively, but not exclusively, HFCP could lead to proteasome inhibition, hence in the accumulation of ubiquitinated proteins (Fig. 9). This accumulation may activate caspase-3-dependent apoptosis, as observed in several cell types [38,39]. Nevertheless, caspase-3 may also be ubiquitinated [39]; thus, its activity may be partially inhibited. This may explain why we observed high molecular bands for caspase-3 on the western blot of cells incubated with HFCP. Moreover, the accumulation of ubiquitinated proteins in cells with proteasome inhibition activates the degradation pathway of autophagy, allowing the cell to resist for some time [40]. However, as homeostasis is not restored, it becomes lethal to HepG2 cells, whereas A549 cells have no “choice” but to switch to apoptosis, perhaps via caspase-8 activation [29]. We thus hypothesize that HFCP may lead to proteasome inhibition, which initiates autophagy as a compensatory mechanism to cope with the accumulation of ubiquitinated proteins, leading to caspase-8 activation and atypical apoptosis.

HFCP exerted cytotoxic effects towards two different cancer cell lines but also in MCF10A cells. MCF10A cells are an immortalized, non-transformed epithelial cell line derived from human fibrocystic mammary tissue. They are often considered as a normal control for cancer cells. It has to be noted that in our culture conditions, etoposide, a chemotherapeutic drug presently used in clinics for treating several types of cancer, also induced a marked decrease in MCF10A viability. More investigations are thus needed to delineate whether the cytotoxic effects of HFCP would be specific to cancer cells or not.

Further characterization of the bioactive pectin fragments and associated biological responses could be of great interest. Because some cancers show resistance to chemotherapeutic treatment, which induces caspase-dependent apoptosis, a new therapeutic treatment that promotes a caspase-independent cell death pathway could enhance the efficiency of current cancer treatments.

## Supporting Information

S1 Fig Heat-modified citrus pectin cytotoxicity. HepG2 and A549 cells were incubated with medium alone (Ctl-), 50 μM etoposide (etop) or hydrolysed citrus pectin at different concentrations. Cell viability was assessed with MTT assay after 24h of incubation. Data are means of triplicates +/− SD (n = 3). *, ** or ***: p< 0.05, p< 0.01 or p< 0.001 using ANOVA I and Tukey’s multiple comparison test.
(PDF)

S2 Fig Heat-modified citrus pectin effects on cell morphology. HepG2 and A549 cells were incubated with medium alone (Ctl-), 50 μM etoposide (etop), 3 mg/ml hydrolysed citrus pectin (HFCP) or 3 mg/ml pectin for 24h and 48h. Micrographs were taken in phase contrast microscopy (objective 20x) after 24h or 48h of incubation.
(PDF)

S3 Fig Effect of serum on heat-modified citrus pectin cytotoxicity. HepG2 cells were incubated with medium alone (Ctl), 1 μM staurosporine (STS), 50 μM etoposide (Etop), different concentrations of hydrolysed citrus pectin (HFCP) or 3 mg/ml citrus pectin (Pectin), with (**A**) or without (**B**) 10% fœtal calf serum. Cell viability was assessed using a MTT assay after 24h of incubation. Data are means of triplicates +/− SD (n = 3). ***: p< 0.001 using ANOVA I and Tukey’s multiple comparison test.
(PDF)

S4 Fig Cytotoxic effect of heat modified citrus pectin at low doses. HepG2 cells were incubated with medium alone (Ctl), 1 μM staurosporine (STS), 50 μM etoposide (Etop), different concentrations of hydrolysed citrus pectin (HFCP) or 3 mg/ml citrus pectin (Pectin), with (**A**) or without (**B**) 10% fœtal calf serum. Cell viability was assessed using a MTT assay after 72h of incubation. Data are means of triplicates +/− SD (n = 3). ***: p< 0.001 using ANOVA I and Tukey’s multiple comparison test.
(PDF)

S5 Fig Cytotoxic effects of heat modified citrus pectin on MCF10A cells. MCF10A cells were incubated with medium alone (Ctl), 1 μM staurosporine (STS), 50 μM etoposide (Etop), different concentrations of hydrolysed citrus pectin (HFCP) or 3 mg/ml citrus pectin (Pectin), with (**A, B**) or without (**C, D**) 10% fœtal calf serum. Cell viability was assessed using a MTT assay after 24h (**A, C**) or 72h (**B, D**) of incubation. Data are means of triplicates +/− SD (n = 3). ***: p< 0.001 using ANOVA I and Tukey’s multiple comparison test.
(PDF)

S6 Fig Cytotoxicity of heat-modified citrus pectin in MCF7 cells. MCF7 cells were incubated with medium alone (Ctl), 50 μM etoposide (etop), 3 mg/ml hydrolysed citrus pectin (HFCP) or 3 mg/ml citrus pectin (Pectin). Cell viability was assessed using a MTT assay after 24h and 48h of incubation. Data are means of triplicates +/− SD (n = 3). ***: p< 0.001 using ANOVA I and Tukey’s multiple comparison test.
(PDF)

S1 Table Effect of Z-VAD-fmk on caspase activity. HepG2 and A549 cells were incubated with medium alone (Ctl-), 50 μM etoposide (Etop), 3 mg/ml hydrolyzed citrus pectin (HFCP) or 3 mg/ml citrus pectin (Pectin), in the presence or in the absence of Z-VAD-fmk at 20 μM, a caspase inhibitor. Caspase activity was measured with MTT assay after different incubation times. Data are means of triplicates +/−SD (n = 3). Statistical analyses were performed were Holm-Sidak test and ANOVAII test. P value in comparison to the corresponding sample without Z-VAD-fmk are *: P ≤ 0.05; ***: P ≤ 0.001.
(PDF)
