# Supplementary material for: Heat-Modified Citrus Pectin Induces Apoptosis-Like Cell Death and Autophagy in HepG2 and A549 Cancer Cells
Source: PLoS One. 2015 Mar 20;10(3):e0115831. doi: 10.1371/journal.pone.0115831 (PMC4368604; doi:10.1371/journal.pone.0115831)
Supplement: S1 Fig — HepG2 and A549 cells were incubated with medium alone (Ctl-), 50 μM etoposide (etop) or hydrolysed citrus pectin at different concentrations. Cell viability was assessed with MTT assay after 24h of incubation. Data are means of triplicates +/− SD (n = 3). *, ** or ***: p< 0.05, p< 0.01 or p< 0.001 using ANOVA I and Tukey’s multiple comparison test. (PDF) [file pone.0115831.s001.pdf]

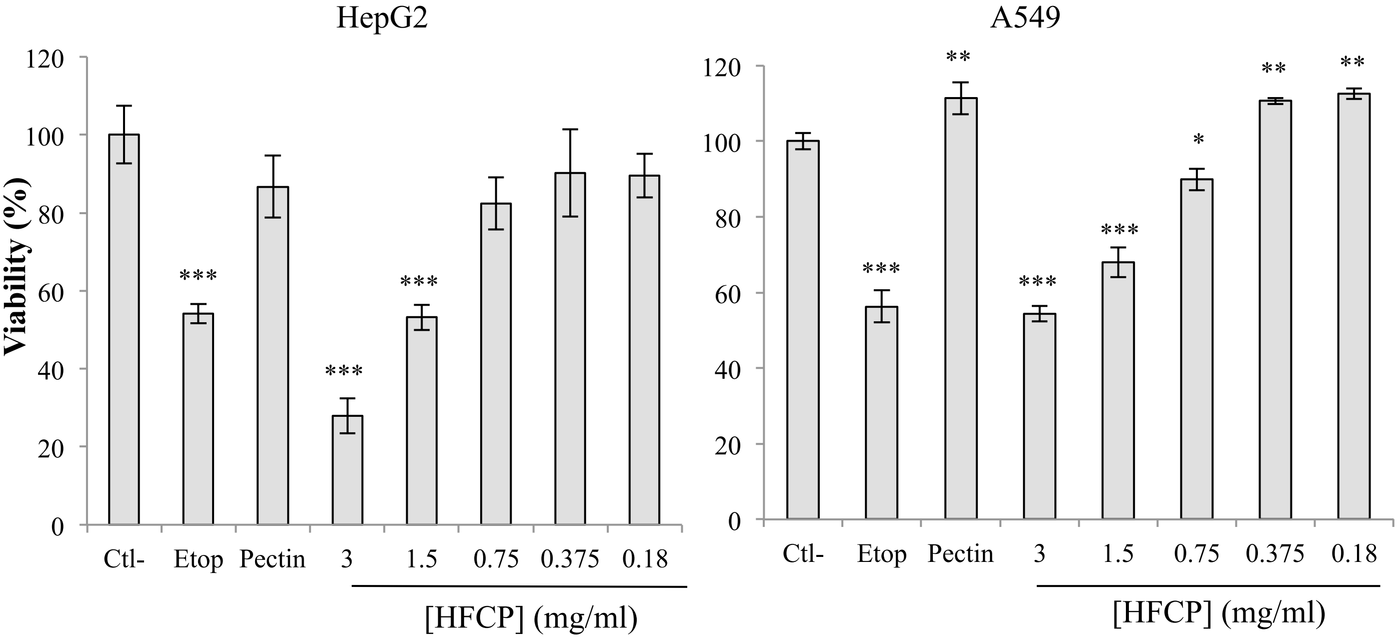

Fig. S1: Heat modified citrus pectin cytotoxicity. HepG2 and A549 cells were incubated with medium alone (Ctl-), 50 µM etoposide (etop) or hydrolysed citrus pectin at different concentrations. Cell viability was assessed with MTT assay after 24h of incubation. Data are means of triplicates +/- SD (n=3). *, ** or *** : $p< 0.05$, $p< 0.01$ or $p< 0.001$ using ANOVA I and Tukey's multiple comparison test.
