# Supplementary material for: Heat-Modified Citrus Pectin Induces Apoptosis-Like Cell Death and Autophagy in HepG2 and A549 Cancer Cells
Source: PLoS One. 2015 Mar 20;10(3):e0115831. doi: 10.1371/journal.pone.0115831 (PMC4368604; doi:10.1371/journal.pone.0115831)
Supplement: S2 Fig — HepG2 and A549 cells were incubated with medium alone (Ctl-), 50 μM etoposide (etop), 3 mg/ml hydrolysed citrus pectin (HFCP) or 3 mg/ml pectin for 24h and 48h. Micrographs were taken in phase contrast microscopy (objective 20x) after 24h or 48h of incubation. (PDF) [file pone.0115831.s002.pdf]

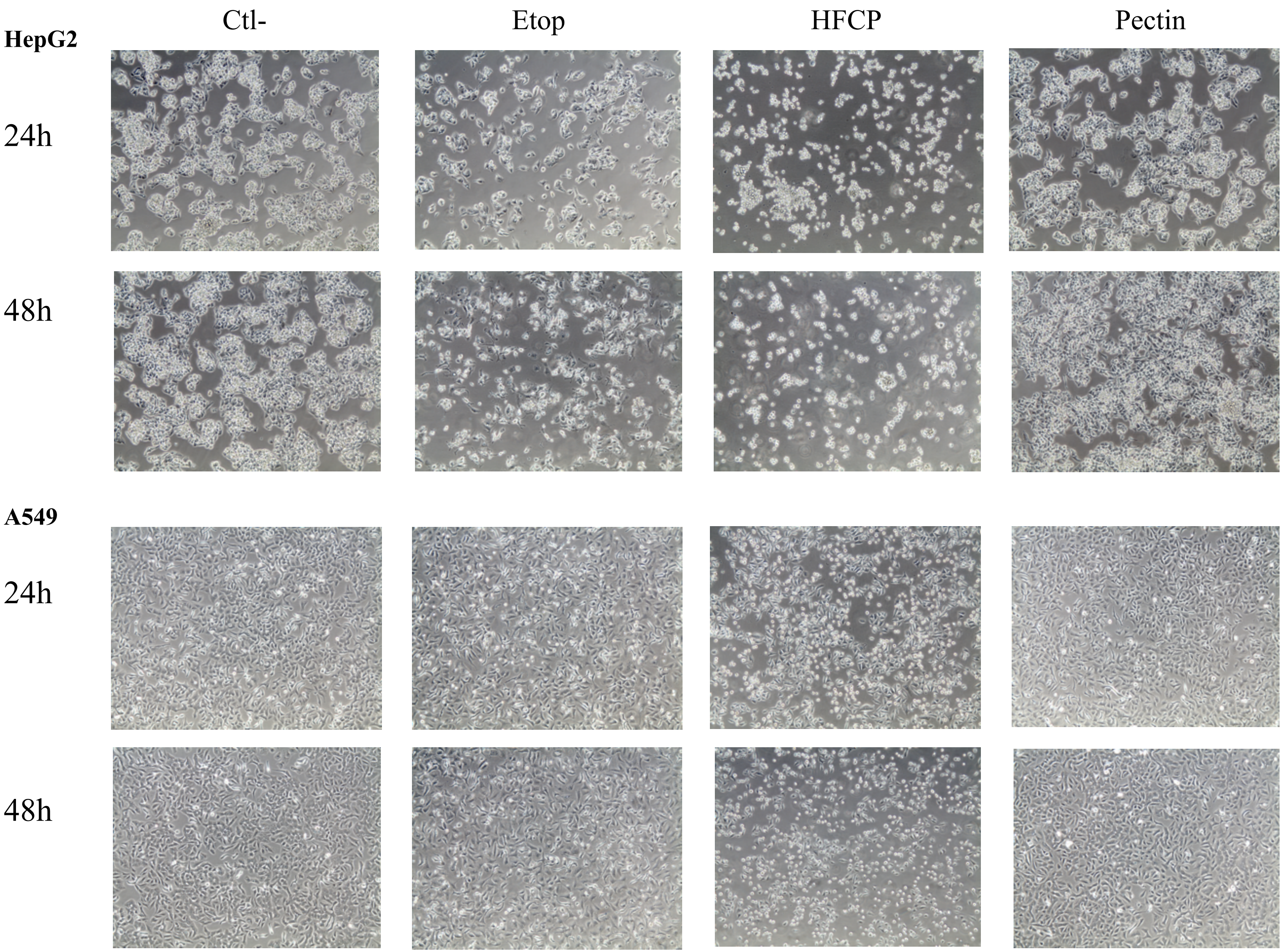

Fig. S2: Heat modified citrus pectin effects on cell morphology. HepG2 and A549 cells were incubated with medium alone (Ctl-), 50 μM etoposide (etop), 3 mg/ml hydrolysed citrus pectin (HFCP) or 3 mg/ml pectin for 24h and 48h. Micrographs were taken in phase contrast microscopy (objective 20x) after 24h or 48h of incubation.
