# Supplementary material for: Heat-Modified Citrus Pectin Induces Apoptosis-Like Cell Death and Autophagy in HepG2 and A549 Cancer Cells
Source: PLoS One. 2015 Mar 20;10(3):e0115831. doi: 10.1371/journal.pone.0115831 (PMC4368604; doi:10.1371/journal.pone.0115831)
Supplement: S3 Fig — HepG2 cells were incubated with medium alone (Ctl), 1 μM staurosporine (STS), 50 μM etoposide (Etop), different concentrations of hydrolysed citrus pectin (HFCP) or 3 mg/ml citrus pectin (Pectin), with (A) or without (B) 10% fœtal calf serum. Cell viability was assessed using a MTT assay after 24h of incubation. Data are means of triplicates +/− SD (n = 3). ***: p< 0.001 using ANOVA I and Tukey’s multiple comparison test. (PDF) [file pone.0115831.s003.pdf]

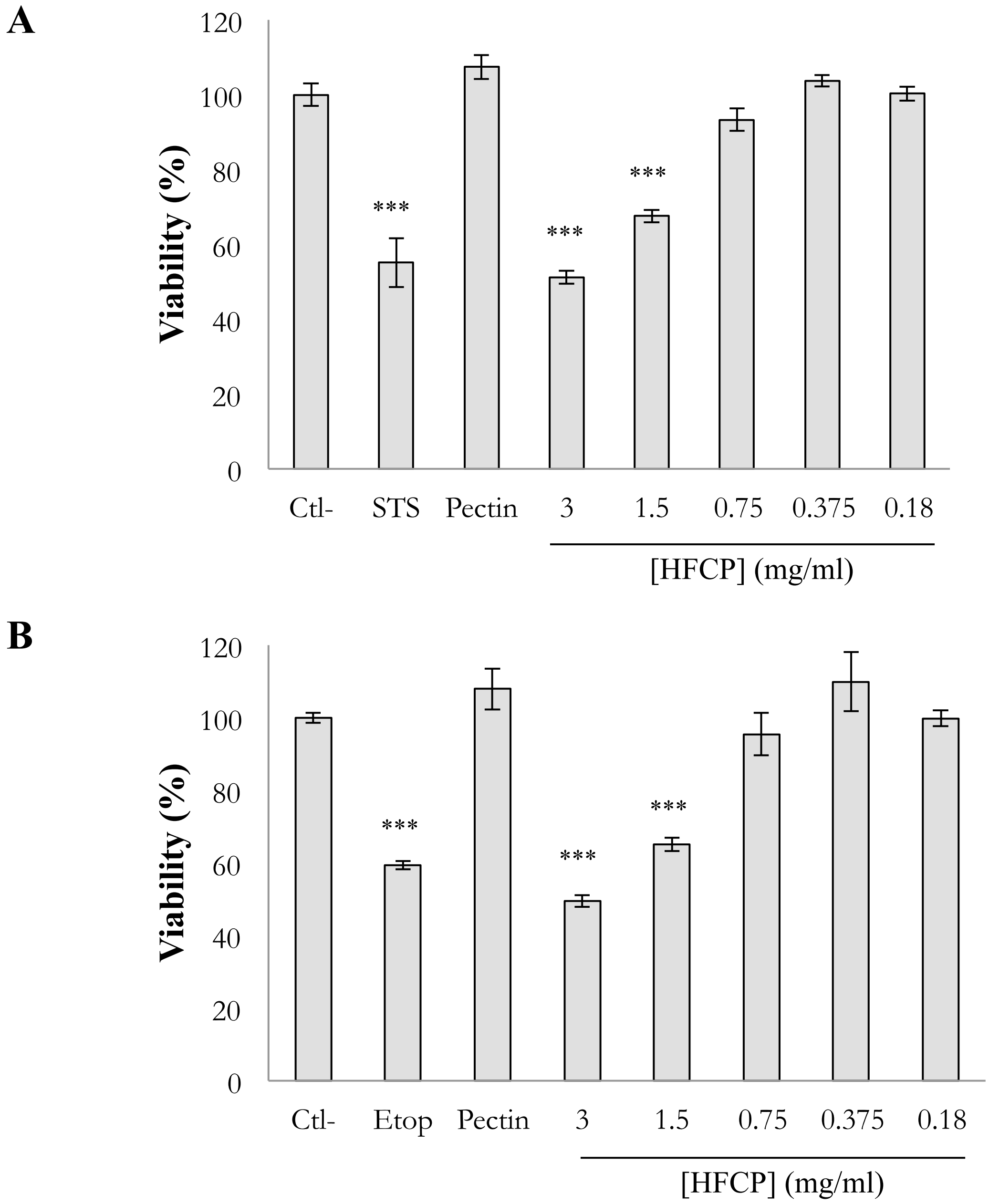

Fig. S3: Effect of serum on heat modified citrus pectin cytotoxicity. HepG2 cells were incubated with medium alone (Ctl), 1 µM staurosporine (STS), 50 µM etoposide (Etop), different concentrations of hydrolysed citrus pectin (HFCP) or 3 mg/ml citrus pectin (Pectin), with **(A)** or without **(B)** 10% fœtal calf serum. Cell viability was assessed using a MTT assay after 24h of incubation. Data are means of triplicates +/- SD (n=3). . *** : $p < 0.001$ using ANOVA I and Tukey's multiple comparison test.
