# Supplementary material for: Heat-Modified Citrus Pectin Induces Apoptosis-Like Cell Death and Autophagy in HepG2 and A549 Cancer Cells
Source: PLoS One. 2015 Mar 20;10(3):e0115831. doi: 10.1371/journal.pone.0115831 (PMC4368604; doi:10.1371/journal.pone.0115831)
Supplement: S5 Fig — MCF10A cells were incubated with medium alone (Ctl), 1 μM staurosporine (STS), 50 μM etoposide (Etop), different concentrations of hydrolysed citrus pectin (HFCP) or 3 mg/ml citrus pectin (Pectin), with (A, B) or without (C, D) 10% fœtal calf serum. Cell viability was assessed using a MTT assay after 24h (A, C) or 72h (B, D) of incubation. Data are means of triplicates +/− SD (n = 3). ***: p< 0.001 using ANOVA I and Tukey’s multiple comparison test. (PDF) [file pone.0115831.s005.pdf]

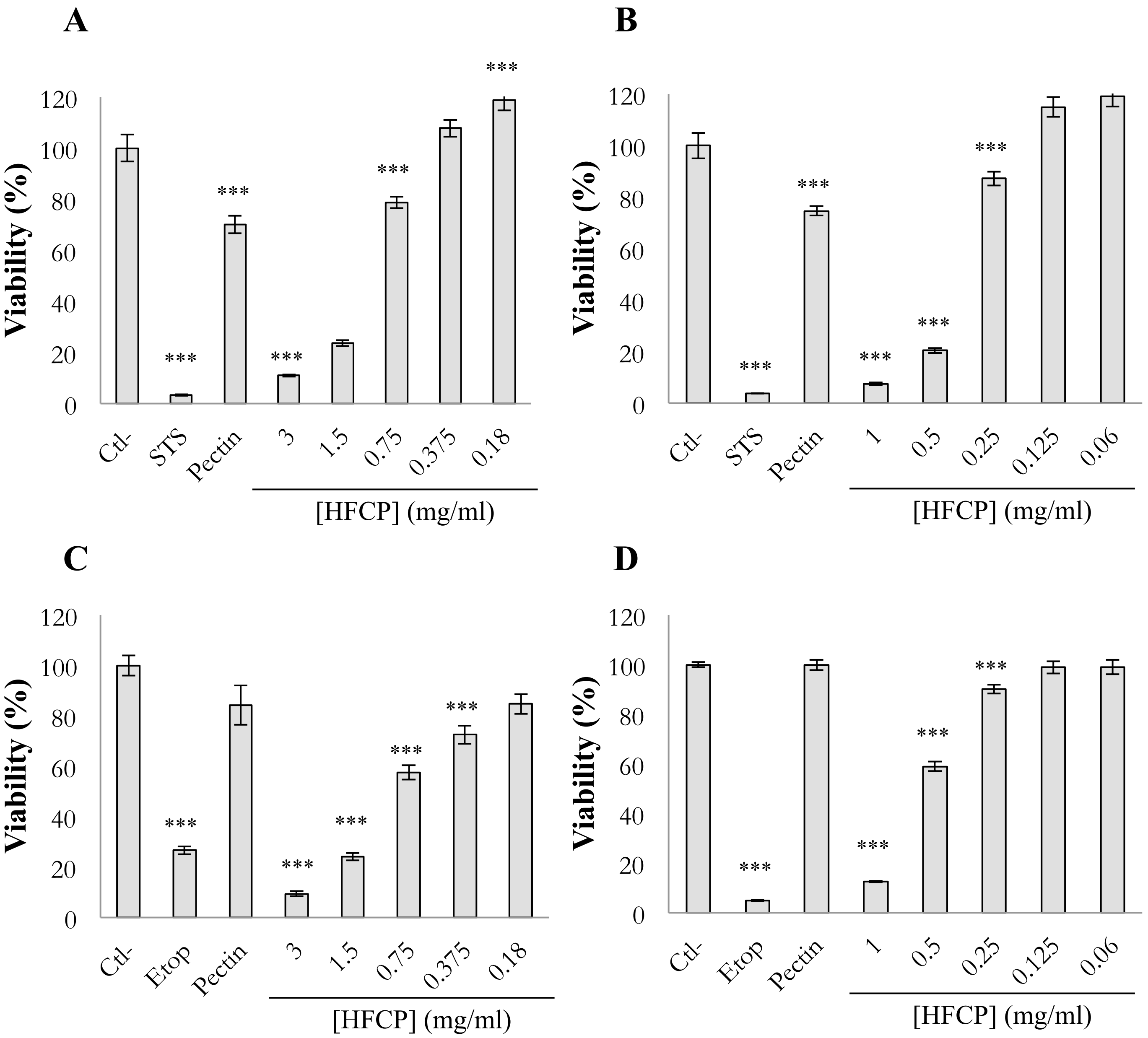

Fig. S5: Cytotoxic effects of heat modified citrus pectin on MCF10A cells. MCF10A cells were incubated with medium alone (Ctl), 1 µM staurosporine (STS), 50 µM etoposide (Etop), different concentrations of hydrolysed citrus pectin (HFCP) or 3 mg/ml citrus pectin (Pectin), with **(A, B)** or without **(C, D)** 10% fœtal calf serum. Cell viability was assessed using a MTT assay after 24h **(A, C)** or 72h **(B, D)** of incubation. Data are means of triplicates +/- SD (n=3). . *** : p< 0.001 using ANOVA I and Tukey's multiple comparison test.
