# Supplementary material for: Heat-Modified Citrus Pectin Induces Apoptosis-Like Cell Death and Autophagy in HepG2 and A549 Cancer Cells
Source: PLoS One. 2015 Mar 20;10(3):e0115831. doi: 10.1371/journal.pone.0115831 (PMC4368604; doi:10.1371/journal.pone.0115831)
Supplement: S1 Table — HepG2 and A549 cells were incubated with medium alone (Ctl-), 50 μM etoposide (Etop), 3 mg/ml hydrolyzed citrus pectin (HFCP) or 3 mg/ml citrus pectin (Pectin), in the presence or in the absence of Z-VAD-fmk at 20 μM, a caspase inhibitor. Caspase activity was measured with MTT assay after different incubation times. Data are means of triplicates +/−SD (n = 3). Statistical analyses were performed were Holm-Sidak test and ANOVAII test. P value in comparison to the corresponding sample without Z-VAD-fmk are *: P ≤ 0.05; ***: P ≤ 0.001. (PDF) [file pone.0115831.s007.pdf]

Table S1: Effect of Z-VAD-fmk on caspase activity

Incubation time	Treatment	HepG2 cells	HepG2 cells + Z-VAD-fmk	A549 cells	A549 cells + Z-VAD-fmk
6 hours	Ctl-	8.5 ± 3.5	3 ± 2.3	14.3 ± 5.5	5.8 ± 3.1
	Etop	16.6 ± 12.8	4.7 ± 4*	59.4 ± 8.5	10.3 ± 9.3***
	HFCP	8.1 ± 4.1	3.7 ± 0.7	21 ± 5	11.2 ± 5.8
	Pectin	14 ± 3.5	3.9 ± 1*	11.9 ± 4.6	10 ± 2.8
24 hours	Ctl-	18.9 ± 5.6	3.8 ± 2.4	27.2 ± 7.1	5.2 ± 1.4
	Etop	153.9 ± 39.6	4 ± 1.6***	143 ± 35.6	5.8 ± 2.4***
	HFCP	138.6 ± 37.9	7.3 ± 6.5***	53.7 ± 5.7	5.8 ± 4.9***
	Pectin	12.7 ± 0.7	7.5 ± 4.5	21.1 ± 5.7	5.8 ± 2.9
48 hours	Ctl-	40.4 ± 15.1	2.6 ± 0.4	25.9 ± 6.3	7.9 ± 3.4
	Etop	30.1 ± 2	2.9 ± 3.9	85.9 ± 46.9	4 ± 3***
	HFCP	313 ± 69	3 ± 2.2***	77.5 ± 5.1	5.9 ± 3.6***
	Pectin	39.7 ± 16.3	6.7 ± 1	44.3 ± 17.5	6.8 ± 3.4*

HepG2 and A549 cells were incubated with medium alone (Ctl-), 50 µM etoposide (Etop), 3 mg/ml hydrolyzed citrus pectin (HFCP) or 3 mg/ml citrus pectin (Pectin), in the presence or in the absence of Z-VAD-fmk at 20 µM, a caspase inhibitor. Caspase activity was measured with MTT assay after different incubation times. Data are means of triplicates +/-SD (n=3). Statistical analyses were performed were Holm-Sidak test and ANOVAII test. P value in comparison to the corresponding sample without Z-VAD-fmk are *: $P \leq 0.05$; ***: $P \leq 0.001$.
